# Involvement of human and canine MRP1 and MRP4 in benzylpenicillin transport

**DOI:** 10.1371/journal.pone.0225702

**Published:** 2019-11-27

**Authors:** Xiaofen Zhao, Yangfang Li, Kun Du, Yuqin Wu, Ling Liu, Shan Cui, Yan Zhang, Jin Gao, Richard F. Keep, Jianming Xiang

**Affiliations:** 1 Department of Neonate, Kunming Children’s Hospital, Kunming, China; 2 Department of Neurosurgery, University of Michigan Medical School, Ann Arbor, Michigan, United States of America; Hungarian Academy of Sciences, HUNGARY

## Abstract

The blood-brain barrier (BBB) is a dynamic and complex interface between blood and the central nervous system (CNS). It protects the brain by preventing toxic substances from entering the brain but also limits the entry of therapeutic agents. ATP-binding cassette (ABC) efflux transporters are critical for the functional barrier and present a formidable impediment to brain delivery of therapeutic agents including antibiotics. The aim of this study was to investigate the possible involvement of multidrug resistance-associated protein 1 and 4 (MRP1 and MRP4), two ABC transporters, in benzylpenicillin efflux transport using wild-type (WT) MDCKII cells and cells overexpressing those human transporters, as well as non-selective and selective inhibitors. We found that inhibiting MRP1 or MRP4 significantly increased [^3^H]benzylpenicillin uptake in MDCKII-WT, -MRP1 or –MRP4 cells. Similar results were also found in HepG2 cells, which highly express MRP1 and MRP4, and hCMEC/D3 cells which express MRP1. The results indicate that human and canine MRP1 and MRP4 are involved in benzylpenicillin efflux transport. They could be potential therapeutic targets for improving the efficacy of benzylpenicillin for treating CNS infections since both MRP1 and MRP4 express at human blood-brain barrier.

## Introduction

Treating CNS disorders is a huge challenge because of the presence of the blood-brain barrier (BBB) which is a dynamic physical and biological barrier between blood circulation and the central nervous system (CNS). The unique features of the BBB lie in the structure/function of the cerebral microvascular endothelial cells and the neurovascular unit comprised of those cells and surrounding astrocytes, pericytes and extracellular matrix. It offers a unique protection to CNS by restricting the entry of toxin, pathogen and xenobiotics into brain and, at same time, it limits the delivery of therapeutic agents to the brain[1–3]. Unlike other organs of the human body, more than 98% of small molecules and almost 100% of large therapeutic molecules cannot reach the brain via the circulatory system. ABC (ATP-binding cassette) efflux transporters, expressed on the luminal (blood-facing) plasma membrane of brain capillary endothelial cells, are an important functional part of the BBB. They play a critical role in keeping drugs and neurotoxic substances from entering the brain and in transporting toxic metabolites out of the brain[4–6]. ABC efflux transporters include P-gp (P-glycoprotein), BCRP (breast cancer resistance protein) and MRPs (multidrug resistance proteins, ABCCs; which have 13 members), are known to be involved in exporting a wide range of drugs, such as antibiotics, anti-HIV drugs, anticancer agents, antihistamines, immunosuppressive drugs and analgesics, at the BBB[7–14]. They are a potential target and an innovative strategy in treating CNS diseases and protecting brain since changes in the transporter expression and transport activity can have a major effect on pharmacotherapy[15–19].

Beta-lactam antibiotics are a class of drugs consisting of all antibiotic agents that contain a beta-lactam ring in their molecular structure. This includes penicillins, cephalosponins, cephamycins, carbapenems and monobactams. Because of their wide spectrum and broad therapeutic index, they are among the most commonly prescribed antibiotics in treating bacterial infections, including those of the CNS[20, 21]. That includes neonatal purulent meningitis, which has a high mortality rate and causes neurological sequelae and lifelong impairment[22, 23]. Although uncommon, beta-lactam antibiotic toxicity is severe and antibiotic resistance also often develops[24, 25]. Benzylpenicillin penetration across the BBB is limited, but peripherally administered high dose benzylpenicillin can cause seizures[25, 26]. The mechanisms regulating benzylpenicillin entry into brain are still not clear, particularly regarding which ABC transporters may be involved in benzylpenicillin efflux at the human BBB. Previous studies have indicated that some beta-lactam antibiotics, such as benzylpenicillin, ceftriaxone and ampicillin, are substrates of P-gp, which might account for low brain penetration[27–30]. In contradiction, another study suggested that P-gp and BCRP are not involved in benzylpenicillin efflux transport in human[31]. Interestingly, our previous study has shown that benzylpenicillin is a substrate of human BCRP, but not P-gp[32]. However, it has not yet been reported if benzylpenicillin is a substrate of MRPs in human.

The aim of this study was to investigate if MRPs are involved in benzylpenicillin efflux transport in human. We focused on MRP1 and MRP4 because they express in human brain endothelial cells and selective inhibitors for them are commercially available.

## Materials and methods

MDCKII-WT, MDCKII-MRP1 and MDCKII-MRP4 cells were obtained from Netherlands Cancer Institute (Dr. A. H. Schinkel), Amsterdam, Netherlands. Hep G2 cell was purchased from ATCC (Manassas, VA, USA). hCMEC/D3 cells were obtained from Dr. Pierre-Olivier Couraud (Institute Cochin, Paris, France). Cell culture medium and fetal bovine serum (FBS) was purchased from Thermo Fisher Scientific (Grand Island, NY, USA). The non-selective MRP inhibitor MK 571 was from Cayman Chemical (Ann Arbor, MI, USA) and selective MRP1 inhibitor reversan[33, 34] and selective MRP4 inhibitor ceefourin 1[35, 36] were from Sigma (St. Louis, MO, USA) and ABCAM (Cambridge, MA, USA). [^3^H]Benzylpenicillin (25 Ci/mmol) and [^14^C]mannitol (55 mCi/mmol) were purchased from American Radiolabeled Chemicals, Inc. (St. Louis, MO, USA).

### Cell culture

For [^3^H]benzylpenicillin uptake experiments, MDCKII-WT, MDCKII-MRP1, MDCKII-MRP4 and Hep G2 were cultured in 12-well plates. As previously described[37], cells were grown in DMEM supplemented with 10% FBS in humidified incubator with 95% air-5% CO2 at 37°C and the medium was changed every other day. hCMEC/D3 cells were cultured in DMEM-F12 supplemented with: 10% FBS, 15mM Hepes, 2mM glutamine, insulin: 5μg/ml, EGF: 50ng/ml EGF, bFGF: 25ng/ml, hydrocortisone: 2μg/ml and transferrin: 5μg/ml. hCMEC/D3 were also grown in humidified incubator with 95% air-5% CO2 at 37°C and the medium was changed every other day. All of cells were ready for uptake experiment when they reached 80–90% confluency.

### [^3^H]Benzylpenicillin uptake

[^3^H]Benzylpenicillin uptake was examined in MDCKII-WT, MDCKII-MRP1,MDCKII-MRP4, Hep G2 and hCMEC/D3 cells. The culture medium was removed at the beginning of the experiment and the cells were washed once with DMEM. Then, 1ml of uptake medium (DMEM) containing 0.1μCi [^3^H]benzylpenicillin and 0.05μCi [^14^C]mannitol (internal control), with or without inhibitors, was added to initiate uptake. After incubating at 37°C for 1 hour[32], the uptake medium was removed and the cells were rapidly washed with ice-cold PBS for three times. Hyamine hydroxide was used to lyse the cells and the cell lysis was counted in a liquid scintillation counter (Beckman Coulter LS6500). Uptake was expressed per mg cell protein and [^14^C]mannitol (about 5% of [^3^H]Benzylpenicillin in the cells) was used to correct for extracellular contamination as described previously[32].

### Quantitative real time RT-PCR (qRT-PCR)

All of materials were from Thermo Fisher Scientific (Grand Island, NY, USA), unless otherwise stated. Total RNA was extracted from the cells using TRIzol Reagent following the manufacturer’s instructions. The concentration and purity of RNA were measured spectrophotometrically at 260 and 280 nm with NanoDrop 2000 (Thermo Fisher Scientific). cDNA was synthesized from 1μg of total RNA in a 20μl reaction mixture using a High Capacity cDNA Reverse Transcription Kit with RNase Inhibitor. The mixture was incubated at 25°C for 10 minutes, 37°C for 120 minutes, and 85°C for 5 minutes. Veriquest SYBR green master mix was used for Real-time PCR in an Eppendorf Thermal Cycler. The reaction mixture was incubated at 95°C for 10 minutes and cycled 40 times from 95°C for 15 seconds to 60°C for 1 minute. The primers used for qRT-PCR are shown in Table 1. All PCR data were normalized to GAPDH using the Double Delta CT Value method.

**Table 1:** Primer sequences for qRT-PCR.

Genes	Accession #	Forward (5’—3’)	Reverse (5’—3’)
MRP1, canine	NM_001002971.1	GGCTCTGCTTCCCCTTCTAC	GGATTTTGCCCCAACTTCTT
MRP4, canine	NM_001197174.1	ATTCTGTGGCTCTGCACGAA	GGTTGACAATCTGGCCGGTA
GAPDH, canine	AB038240.1	GCGGGGCCAAGAGGGTCATCAT	GCTTTCTCCAGGCGGCAGGTCAG
MRP1, human	L05628.1	CTCCCCGGTCTATTCCCATTTCAA	TCTCGGTAGCGCAGGCAGTAGTTC
MRP4 , human	AY207008.1	GTGTACCAGGAGGTGAAGCC	CCTCTCCAAGGTGCTGTGAG
GAPDH, human	BC023632.2	GGGGAGCCAAAAGGGTCATCATC	GACGCCTGCTTCACCACCTTCTTG

### Statistical analysis

All measurements were performed in triplicate wells and data collected from at least three independent experiments are presented as means ± SEM. Results were analyzed by t-test or one-way analysis of variance (ANOVA) followed by Dunnett *post hoc* test for comparisons to a single control or Tukey *post hoc* test for multiple comparisons between groups. A *P* value less than 0.05 was considered statistically significant (*P< 0.05, **P< 0.01 and ***P< 0.001). All analyses were performed with Prism 7 (Graph Pad, San Diego, CA).

## Results

**Fig 1:**
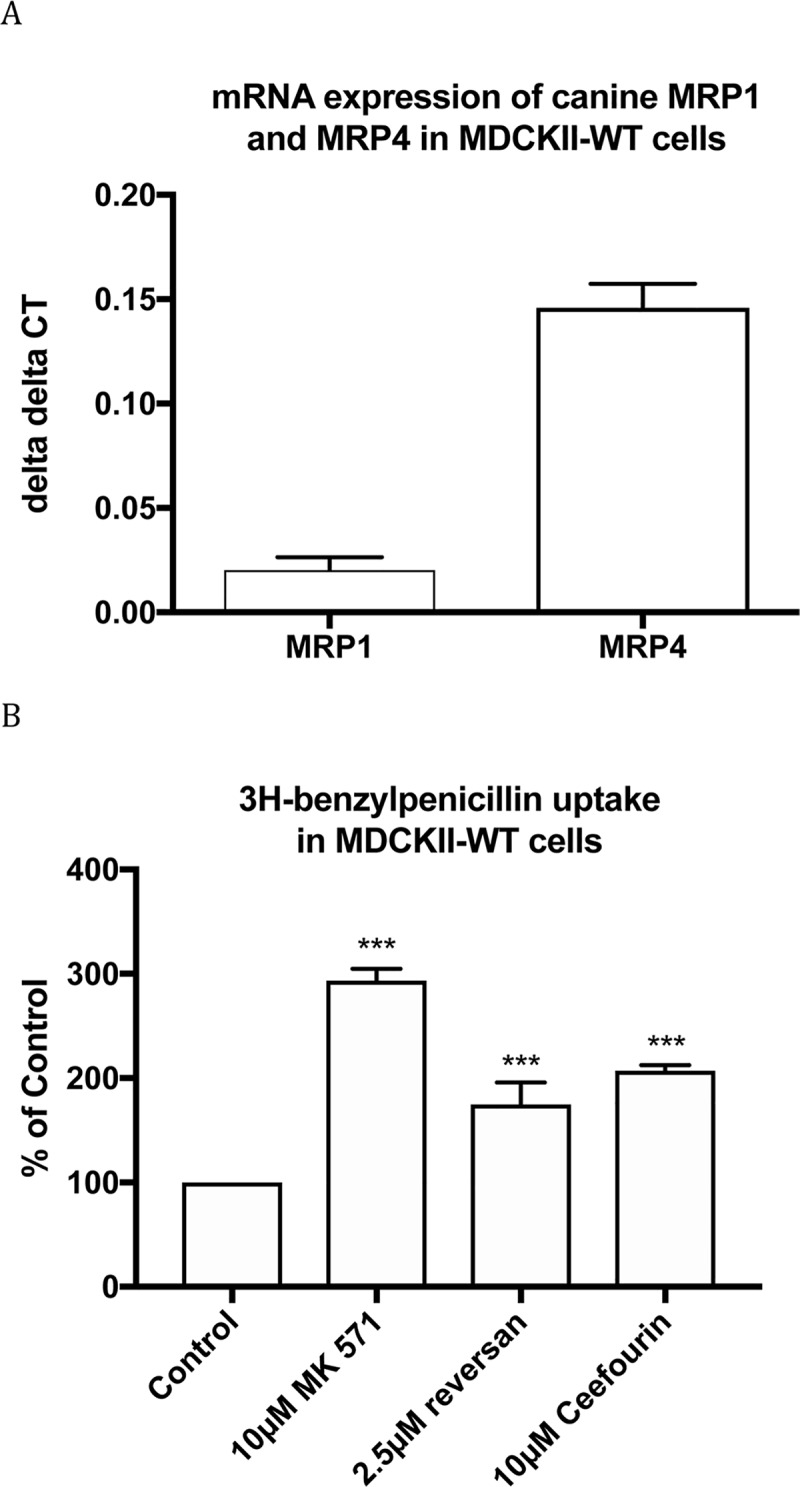
mRNA expression of MRP transporters in MDCKII-WT cells (A) and [^3^H]benzylpenicillin uptake in MDCKII-WT cells with and without inhibitors (B). Values are means +/- S.E., n = 3, each performed in triplicate. *** indicate significant differences from comparison between different cells at the P<0.001 level.

Wild-type MDCKII (WT) is a canine epithelial cell line which expresses MRP1, MRP2 and MRP5[38] and our qRT-PCR data showed that it also expresses MRP4 (Fig 1A). The cellular uptake of [^3^H]benzylpenicillin was examined with and without non-selective MRP inhibitor (MK571), selective MRP1 inhibitor (reversan) and MRP4 inhibitor (ceefourin 1). Compared to control, 10μM MK571, 2.5μM reversan and 10μM ceefourin 1 significantly increased [^3^H]benzylpenicillin uptake in MDCKII-WT cells by 2.94±0.06, 1.75±0.12 and 2.07±0.05 fold, respectively (Fig 1B). These results suggest that benzylpenicillin is a MRP1 and MRP4 substrate in these canine cells.

**Fig 2:**
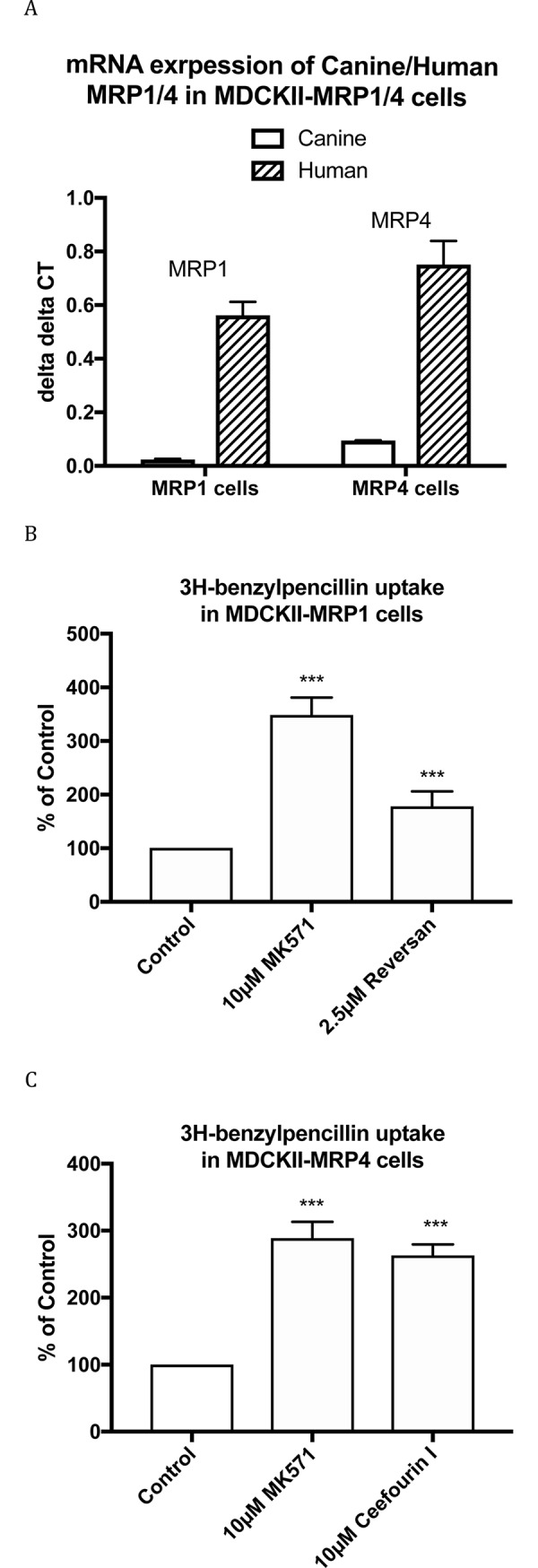
mRNA expression of human and canine MRP1 and MRP4 in MDCKII-MRP1 and–MRP4 cells (A), [^3^H]benzylpenicillin uptake in MDCKII-MRP1 (B) and MDCKII-MRP4 (C) cells with and without inhibitors. Values are means +/- S.E., n = 3–6, each performed in triplicate. *** indicate significant differences from comparison between different cells at the P<0.001 level.

MDCKII-MRP1 and MDCKII-MRP4 are MDCKII cell lines overexpressing human MRP1 and MRP4, which were 23.9 and 8 times higher than canine MRP1 and MRP4, respectively (Fig 2A). As shown in Fig 2B and 2C, [^3^H]benzylpenicillin uptake was increased 2.35±0.18 and 3±0.11 fold by 10μM MK571 and 2.5μM reversan in MDCKII-MRP1 cells, and increased 2.87±0.14 and 2.63±0.09 fold by 10μM MK571 and 10μM ceefourin 1 in MDCKII-MRP4 cells, compared to control, indicating benzylpenicillin is also a substrate of human MRP1 and MRP4.

**Fig 3:**
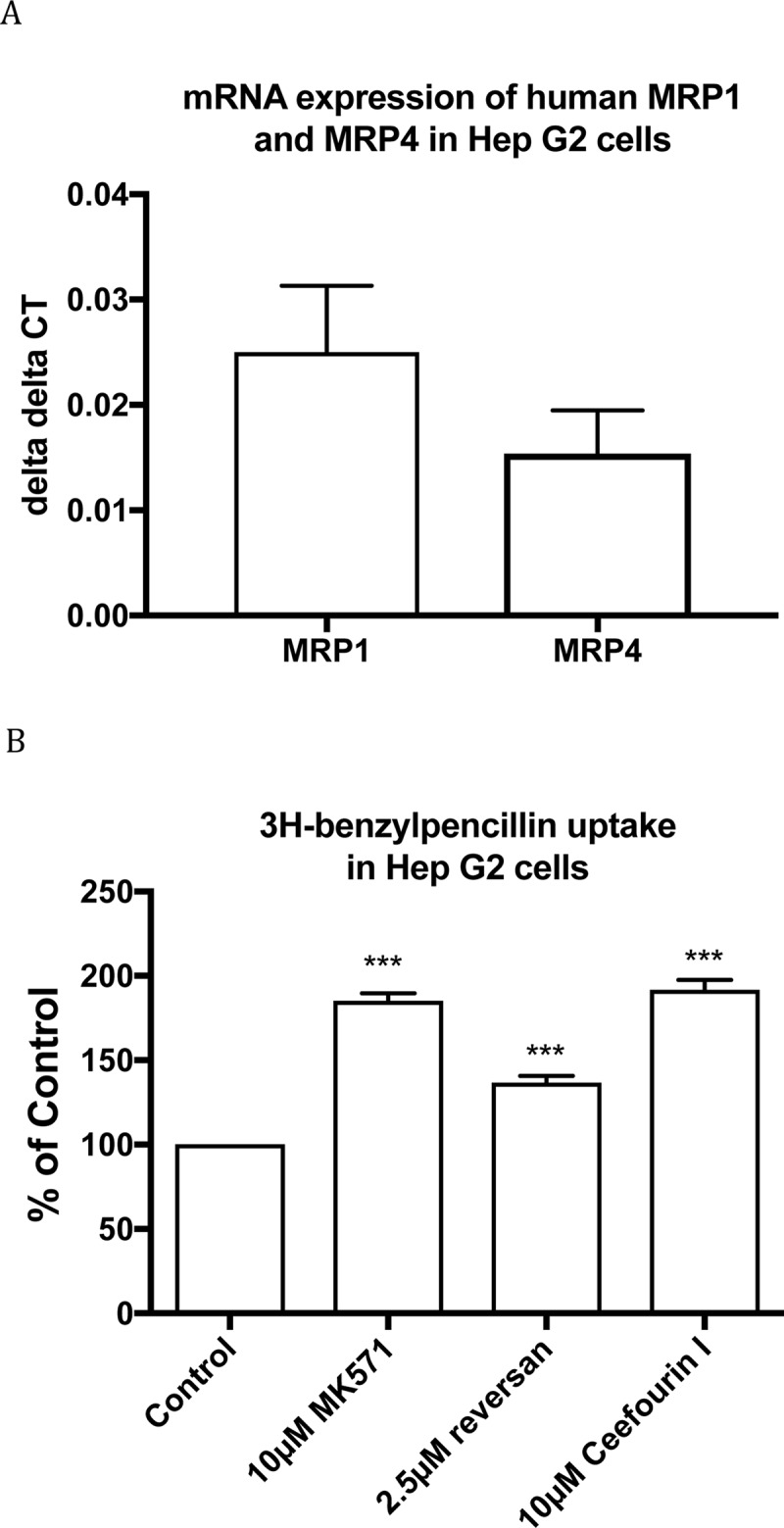
mRNA expression of MRP transporters in Hep G2 cells (A) and [^3^H]benzylpenicillin uptake in Hep G2 cells with and without inhibitors(B). Values are means +/- S.E., n = 3, each performed in triplicate. *** indicate significant differences from comparison between different cells at the P<0.001 level.

Hep G2 is a cell line from human hepatocellular carcinoma. According to prior reports, Hep G2 cells express MRP1, MRP2 and MRP3 [39, 40]. Our qRT-PCR result showed that Hep G2 cells also express MRP4 (Fig 3A). [^3^H]benzylpenicillin uptake in Hep G2 cells was enhanced 1.85±0.03, 1.37±0.02 or 1.92±0.03 fold when 10μM MK571, 2.5μM reversan or 10μM ceefourin 1 was present, respectively (Fig 3B).

**Fig 4:**
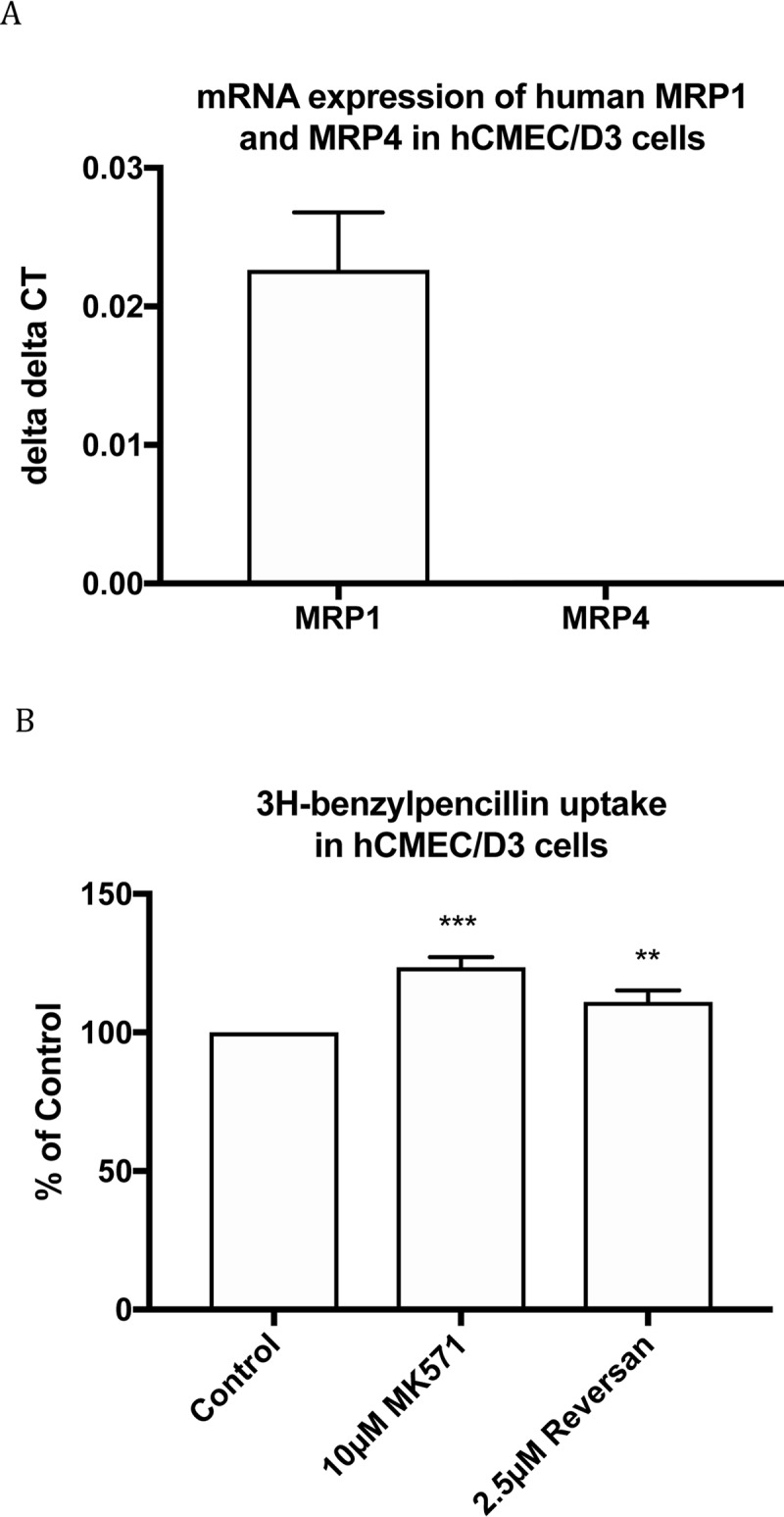
mRNA expression of MRP transporters in hCMEC/D3 cells (A) and [^3^H]benzylpenicillin uptake in hCMEC/D3 cells with and without 10μM MK 571 and 2.5μM reversan(B). Values are means +/- S.E., n = 3–6, each performed in triplicate. ** and *** indicate significant differences from comparison between different cells at the P<0.01 and P<0.001 levels, respectively.

hCMEC/D3 is a human cerebral microvascular endothelial cell line. Prior studies have shown MRP1, MRP2 and MRP4 expression in hCMEC/D3 cells[41, 42]. However, while qRT-PCR assay confirmed MRP1 expression in the current study, MRP4 was not detected (Fig 4A). Compared to control, [^3^H]benzylpenicillin uptake in hCMEC/D3 cells was increased 1.24±0.02 and 1.11±0.02 fold by 10μM MK571 and 2.5μM reversan, respectively (Fig 4B). Overall, results from Hep G2 and hCMEC/D3 cells provided more evidence that benzylpenicillin is a substrate of endogenous human MRP1 and MRP4.

## Discussion

Benzylpenicillin, a beta-lactam antibiotic, has been used to treat bacterial infections, including those of the brain such as neonatal purulent meningitis. ABC efflux transporters P-gp, BCRP and MRPs are abundantly expressed at the BBB. They have a wide range of substrates including many therapeutic drugs, such as antibiotics and anticancer agents, and restrict those drugs from entering brain. It is very controversial and contradictory whether benzylpencillin can enter brain and whether ABC transporters are involved in benzylpenicillin efflux transport. A previous study has suggested that benzylpenicillin enters brain freely [43] but Rousselle et al. found that benzylpenicillin can barely cross BBB and this can be significantly changed after coupling with SynB1 vector[26]. A study by Poelarends et al. have shown that benzylpenicillin and ampicillin are P-gp substrates after examining the effect of LmrA (a structural and functional homolog of human P-gp) expression on the relative antibiotic resistance of E. Coli CS1562[28]. However, we have reported in our previous study that BCRP, but not P-gp, is involved in benzylpenicilin efflux transport in human. Results from the current study showed that inhibiting MRP1 and MRP4 in MDCKII-MRP1 and MDCKII-MRP4 cells with selective or non-selective inhibitors significantly increased benzylpenicillin amount in the cells. Similar results were found in Hep G2 (a cell line from human hepatocellular carcinoma) and hEMEC/D3 (a human cerebral microvascular endothelial cell line) cells. All of the results from the current study indicate that benzylpenicillin is also a substrate of human MRP1 and MRP4.

MRP1 and MRP4 are structurally close and their expression in human brain capillary endothelial cells, astrocytes, microglia and choroid plexus (which form the blood-CSF barrier) are well documented. They have been demonstrated to be associated with brain tumor resistance and also mediate efflux transport of a wide range of drugs such as glutathione, glucuronide, sulfate conjugates, immunosuppressants, HIV protease inhibitors, organic anions, prostaglandins and nucleoside analogs[4, 6]. In this study, we found that that MRP1 and MRP4 are also involved in antibiotic efflux transport.

Among ABC efflux transporters, MRPs are the largest group, consisting of 13 subfamily members [4, 6]. Besides MRP1 and MRP4, MRP2, MRP3 and MRP5 are also expressed in human brain capillary endothelial cells [4]. It is still unknown if other MRP transporters are involved in benzylpenicillin efflux transport, and this needs to be investigated in further studies. Furthermore, all beta-lactam antibiotics have a beta-lactam ring in their molecular structure and it is possible that other beta-lactam antibiotics are also substrates of human MRP1 and MRP4, because of their structural similarity. This also needs further studies.

ABC efflux transporters play a very important role at BBB in protecting the brain but, at the same time, they are an obstacle for therapeutic agents entering the brain. Therefore, there has been great interest in modulating ABC transporters at the BBB in order to increase brain protection (up-regulating ABC transporters) or improve drug delivery to the brain (inhibiting ABC transporters)[18, 19, 44, 45].

In summary, we found in this study that benzylpenicillin is a substrate of MRP1 and MRP4 in human and inhibiting these two transporters, plus BCRP, could be a new strategy to increase benzylpenicillin entering into brain to treat infection.

## Supporting information

S1 Table Data for Fig 1. A) Raw data on mRNA expression of canine MRP1 and MRP4 in MDCKII-WT cells (delta delta CT) used for Fig 1A and 1B) Data of [^3^H]-benzylpenicillin uptake in MDCKII-WT cells (% of Control) used for Fig 1B along with the raw data.
(XLSX)

S2 Table Data for Fig 2. A) Raw data on mRNA expression of Canine/Human MRP1/4 in MDCKII-MRP1/4 cells (delta delta CT) used for Fig 2A. B) Data of [^3^H]-benzylpenicillin uptake in MDCKII-MRP1 cells (% of Control) used for Fig 2B and 2C) Data of [^3^H]-benzylpenicillin uptake in MDCKII-MRP4 cells (% of Control) used for Fig 2C.
(XLSX)

S3 Table Data for Fig 3. A) Raw data on mRNA expression of Human MRP1 and MRP4 in Hep G2 cells (delta delta CT) used for Fig 3A and 3B) Data of [^3^H]-benzylpenicillin uptake in Hep G2 cells (% of Control) used for Fig 3B.
(XLSX)

S4 Table Data for Fig 4. A) Raw data on mRNA expression of Human MRP1 and MRP4 in hCMEC/D3 cells (delta delta CT) used for Fig 4A and 4B) Data of [^3^H]-benzylpenicillin uptake in hCMEC/D3 cells (% of Control) used for Fig 4B.
(XLSX)
